# Pharmacochaperoning in a *Drosophila* model system rescues human dopamine transporter variants associated with infantile/juvenile parkinsonism

**DOI:** 10.1074/jbc.M117.797092

**Published:** 2017-09-29

**Authors:** H. M. Mazhar Asjad, Ameya Kasture, Ali El-Kasaby, Michael Sackel, Thomas Hummel, Michael Freissmuth, Sonja Sucic

**Affiliations:** From the ‡ Institute of Pharmacology and the Gaston H. Glock Research Laboratories for Exploratory Drug Development, Center of Physiology and Pharmacology, Medical University of Vienna, A-1090 Vienna, Austria and; the § Department of Neurobiology, University of Vienna, A-1090 Vienna, Austria

**Keywords:** chaperone, dopamine, dopamine transporter, endoplasmic reticulum (ER), neurotransmitter transport, dopamine

## Abstract

Point mutations in the gene encoding the human dopamine transporter (hDAT, SLC6A3) cause a syndrome of infantile/juvenile dystonia and parkinsonism. To unravel the molecular mechanism underlying these disorders and investigate possible pharmacological therapies, here we examined 13 disease-causing DAT mutants that were retained in the endoplasmic reticulum when heterologously expressed in HEK293 cells. In three of these mutants, *i.e.* hDAT-V158F, hDAT-G327R, and hDAT-L368Q, the folding deficit was remedied with the pharmacochaperone noribogaine or the heat shock protein 70 (HSP70) inhibitor pifithrin-μ such that endoplasmic reticulum export of and radioligand binding and substrate uptake by these DAT mutants were restored. In *Drosophila melanogaster*, DAT deficiency results in reduced sleep. We therefore exploited the power of targeted transgene expression of mutant hDAT in *Drosophila* to explore whether these hDAT mutants could also be pharmacologically rescued in an intact organism. Noribogaine or pifithrin-μ treatment supported hDAT delivery to the presynaptic terminals of dopaminergic neurons and restored sleep to normal length in DAT-deficient (*fumin*) *Drosophila* lines expressing hDAT-V158F or hDAT-G327R. In contrast, expression of hDAT-L368Q in the *Drosophila* DAT mutant background caused developmental lethality, indicating a toxic action not remedied by pharmacochaperoning. Our observations identified those mutations most likely amenable to pharmacological rescue in the affected children. In addition, our findings also highlight the challenges of translating insights from pharmacochaperoning in cell culture to the clinical situation. Because of the evolutionary conservation in dopaminergic neurotransmission between *Drosophila* and people, pharmacochaperoning of DAT in *D. melanogaster* may allow us to bridge that gap.

## Introduction

Transporters of the SLC6 family mediate the Na^+^-dependent cellular uptake of monoamines and other neurotransmitters (GABA, glycine), osmolytes (taurine, betaine), and amino acids (1). Accordingly they are grouped in four subfamilies (2). Point mutations, which have been linked to human diseases, occur in each subfamily. Examples include mutations in the glycine transporter-2 (GlyT2/SLCA5) that give rise to hypereplexia/startle disease (3), in the creatine transporter-1 (CRT1/SCL6A8) that account for ∼5% of male mental retardation (4), and in the dopamine transporter (DAT/SLC6A3)^5^ that lead to a syndrome of infantile/juvenile dystonia or dopamine transporter deficiency syndrome (5–7). The disease is marked by a hyperkinetic movement disorder, which progresses into parkinsonism (7). In many instances, these SLC6 mutations result in retention of the encoded protein in the endoplasmic reticulum, presumably because of defective folding of the protein (8). The clinical phenotype of recessive mutations is readily explained by the loss of function: an ER-retained neurotransmitter transporter does not reach the cell surface; hence, released neurotransmitter or substrate is not cleared, and vesicular stores are not refilled. The dominant mode of genetic transmission can be rationalized by taking into account that these transporters form oligomers. ER export is contingent on oligomerization: transporters, which are deficient in oligomerization, are trapped in the endoplasmic reticulum (9). Accordingly, mutants and transporter fragments retain the wild-type transporter in the endoplasmic reticulum and thus act in a dominant-negative manner (10–13).

Small molecules can assist in protein folding by acting in a manner analogous to proteinaceous chaperones; they bind reversibly to folding intermediates and lower the energy barrier for the transition into a trajectory that is conducive for formation of the fully folded state. Accordingly, these cognate ligands are hence referred to as pharmacochaperones to differentiate them from chemical chaperones, which act in a nonspecific manner (8). The folding trajectory of DAT is not understood, but several folding-deficient mutants of the serotonin transporter (SERT), a monoamine transporter closely related to DAT, have been examined in detail; the available evidence is consistent with the interpretation that the folding trajectory of SERT proceeds through the inward facing conformation (14–16). Ibogaine and its active metabolite noribogaine bind to and stabilize the inward facing state of SERT (17, 18) and are effective pharmacochaperones of folding deficient SERT mutants (14–16). The chaperone/COPII-exchange model predicts that the stalled folding intermediates can be assisted for ER export by manipulating the chaperone or folding sensor machinery (8); these predictions have been verified (14, 15, 19). More recently we employed pharmacochaperoning to rescue a misfolded mutant of drosophila DAT, dDAT-G108Q (20). In the present work, we combined a cellular analysis of disease-causing human DAT mutants with their expression in *Drosophila melanogaster*. Our experiments show that the folding deficit of two specific mutations, *i.e.* hDAT-V158F or hDAT-G327R, can be corrected by pharmacochaperoning. Thus, our observations argue for a therapeutic trial of noribogaine to remedy the neurological deficits in children harboring these mutations.

## Results

### Intracellular retention of DAT mutants associated with infantile/juvenile parkinsonism

**Figure 1.**
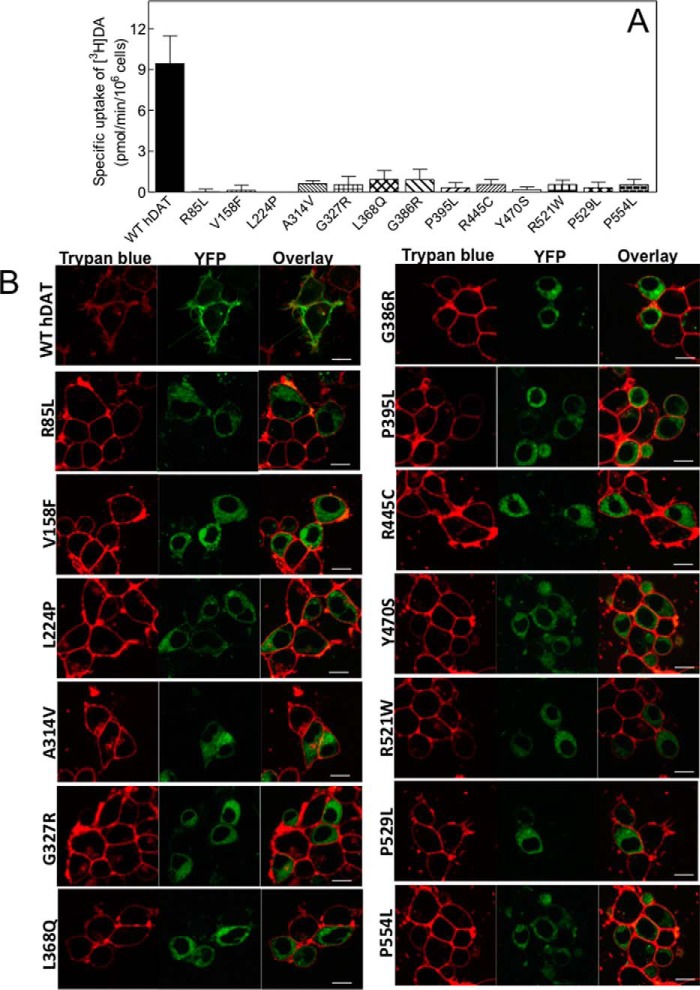
**Human DAT mutants associated with infantile dystonia/parkinsonism generate ER-retained non-functional proteins.** *A*, specific uptake of [^3^H]dopamine was determined as described under “Experimental procedures” in HEK293 cells transiently transfected with plasmids driving the expression of YFP–tagged WT hDAT as a reference and 13 hDAT mutants responsible for infantile dystonia/parkinsonism. *B*, confocal imaging of YFP–tagged WT hDAT and the indicated DAT mutants in transiently transfected HEK293 cells. The transfected cells were seeded onto poly-d-lysine–coated ibidi® glass-bottomed chambers 24 h after transfection. The cells were stained with trypan blue (0.04% in PBS, shown in *red*) to visualize the plasma membrane. The images were captured on a Zeiss LSM710 microscope after an additional 24 h. Overlay images were produced to show co-localization between the different signals. *Scale bars* represent 10 μm.

We expressed 13 DAT mutants that are associated with infantile/juvenile dystonia and parkinsonism (5–7) transiently in HEK293 cells. After transient transfection of plasmids encoding wild-type and mutant transporters, cell numbers were comparable, indicating that the transient expression of the mutated transporters did not affect cell viability. As predicted, the expression of the mutant proteins resulted in [^3^H]dopamine uptake, which was very low when compared with that seen in cells expressing wild-type human DAT (Fig. 1*A*). We also directly verified the intracellular accumulation of DAT mutant by visualizing the cellular distribution of YFP–tagged wild-type and mutant versions of DAT. Images captured by confocal microscopy showed that wild-type DAT was predominantly present at the cell surface. This resulted in extensive co-localization with trypan blue (Fig. 1*B*, *top left panel*), which was used to delineate the cell membrane. In contrast, YFP–tagged DAT mutants were predominantly retained in the cell, and there was little appreciable co-localization with trypan blue.

### Screening of misfolded DAT variants for their response to pharmacochaperoning

**Figure 2.**
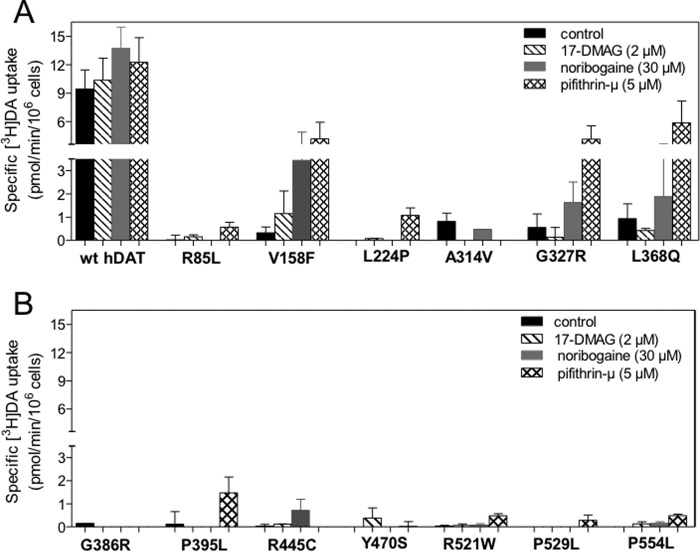
**Screening of hDAT mutants for their response to pharmacochaperoning by noribogaine and the HSP70 inhibitor pifithrin-μ.** *A* and *B*, HEK293 cells were transiently transfected with plasmids driving the expression of YFP–tagged wild-type hDAT and 13 hDAT mutants responsible for infantile dystonia/parkinsonism. After 24 h, the cells were seeded onto 48-well plates for 24 h and treated with 17-DMAG (2 μm), noribogaine (30 μm), or pifithrin-μ (5 μm) for another 24 h. The cells were repeatedly washed to remove the compounds, and uptake of [^3^H]dopamine was subsequently measured as outlined under “Experimental procedures.” Nonspecific uptake was defined as the radioactivity accumulated in the presence of 30 μm mazindole and subtracted. The values for control uptake, *i.e.* uptake in the absence of any pretreatment, are the same as those shown in Fig. 1. The data are from three independent experiments, done in triplicate; the *error bars* indicate S.E.

Trapping of proteins in the ER is indicative of their misfolding; the defective folding can be remedied by various chemical and pharmacological chaperones. We opted for the ibogaine metabolite noribogaine (12-hydroxyibogamine), because for SERT mutants it is a more effective pharmacochaperone than the parent compound ibogaine (14) and because noribogaine rescued hDAT-G140Q and dDAT-G108Q (20). In addition, we also tested pifithrin-μ, a cell-permeable small molecule inhibitor of HSP70 (21) and the HSP90 inhibitor 17-DMAG, because these compounds are predicted to relax control imposed by the heat shock protein relay (8), which operates on the C terminus (14). Accordingly, we preincubated transiently transfected HEK293 cells expressing hDAT mutants associated with infantile/juvenile parkinsonism with noribogaine (30 μm), pifithrin-μ (5 μm), or 17-DMAG (2 μm) for 24 h and subsequently measured dopamine uptake (Fig. 2). After incubation with noribogaine, uptake of dopamine was enhanced in cells expressing DAT-V158F, DAT-G327R, and DAT-L368Q (*gray bars* in Fig. 2). The action of noribogaine was recapitulated by pifithrin-μ (*cross-hatched bars* in Fig. 2); in contrast, the HSP90-inhibitor 17-DMAG was ineffective (*black bars* in Fig. 2). Similarly, 4-phenylbutyric acid (up to 5 mm), which was shown to pharmacochaperone SERT (22), also failed to remedy the folding defects of any of the DAT mutants (data not shown).

**Figure 3.**
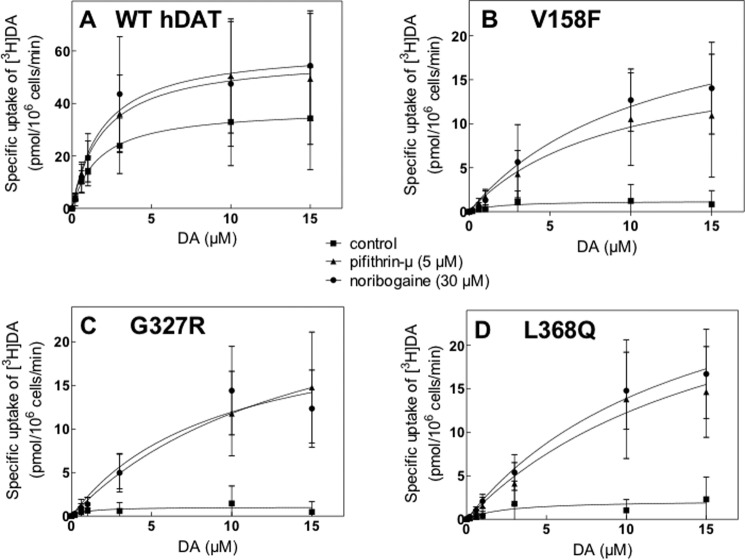
**Kinetics of [^3^H]dopamine uptake by hDAT-V158F, -G327R, and -L368Q before and after pharmacological rescue.** HEK293 cells were transiently transfected with plasmids driving the expression of YFP–tagged wild-type hDAT (*A*), hDAT-V158F (*B*), hDAT-G327R (*C*), and hDAT-L368Q (*D*) were seeded onto 48-well plates. After 24 h, the cells were seeded onto 48-well plates 24 h and incubated in the absence (*squares*) and presence of 30 μm noribogaine (*circles*) and 5 μm pifithrin-μ (*triangles*) for another 24 h. Thereafter, the medium was exchanged, the cells repeatedly washed, and specific [^3^H]dopamine uptake was determined as outlined under “Experimental procedures.” Nonspecific uptake was defined as the radioactivity accumulated in the presence of 30 μm mazindole and subtracted. The data were obtained in four to five independent experiments, done in triplicate; the *error bars* indicate S.D.

Mutations, which interfere with folding, are likely to impede the conformational transitions, which the transporter must achieve to support substrate uptake, even if the folding deficiency is overcome by chaperoning. This was also the case for DAT-V158F (Fig. 3*B*), DAT-G327R (Fig. 3*C*), and DAT-L368Q (Fig. 3*D*); it is evident that the chaperoning action of noribogaine and pifithrin-μ restored the velocity of uptake to substantial levels, but the apparent substrate affinity was reduced, and hence *K_m_* was elevated (Table 1). Pretreatment with noribogaine and pifithrin-μ also enhanced uptake by cells expressing wild-type DAT; this was exclusively due to an increase in *V*_max_ but not to any change in *K_m_* for dopamine (Fig. 3*A* and Table 1). Accordingly, we rule out that the high *K_m_* of DAT-V158F, DAT-G327R, and DAT-L368Q is due to an action of these compounds, *e.g.* residual noribogaine trapping the transporter in the inward facing state and thus affecting the transport cycle.

**Table 1:** **Kinetic parameters for dopamine uptake catalyzed by wild-type DAT, DAT-V158F, DAT-G327R, and DAT-L368Q before and after treatment with pharmacochaperones** The *K_m_* and *V*_max_ values were determined from the data shown in Fig. 3. The values are means ± S.D. from four to five independent experiments performed in triplicate. ND, not determined because of the barely detectable activity in the absence of pharmacochaperones. *K_m_* values of mutant transporters differed significantly from that of the wild-type transporter (*p* < 0.05, Kruskal–Wallis test followed by Dunn's multiple comparison).

	Untreated (control)		Noribogaine-treated		Pifithrin-μ-treated	
	*V*_max_	*K_m_*	*V*_max_	*K_m_*	*V*_max_	*K_m_*
	*pmol*·*10*^−*6*^ *cells*·*min*^−*1*^	μ*m*	*pmol*·*10*^−*6*^ *cells*·*min*^−*1*^	μ*m*	*pmol*·*10*^−*6*^ *cells*·*min*^−*1*^	μ*m*
WT DAT	28.7 ± 6.6	1.5 ± 0.4	56.9 ± 18.1	2.2 ± 0.8	58.1 ± 23.9	1.8 ± 0.7
V158F	ND	ND	20.6 ± 8.8	7.5 ± 4.0	19.7 ± 6.5	8.8 ± 4.9
G327R	ND	ND	30.6 ± 7.9	14.4 ± 9.5	28.3 ± 8.5	10.9 ± 4.0
L368Q	ND	ND	29.5 ± 6.6	12.4 ± 4.1	29.2 ± 8.7	12.6 ± 2.7

### ER export of DAT mutants and their association with heat shock protein 70 after pharmacochaperoning

**Figure 4.**
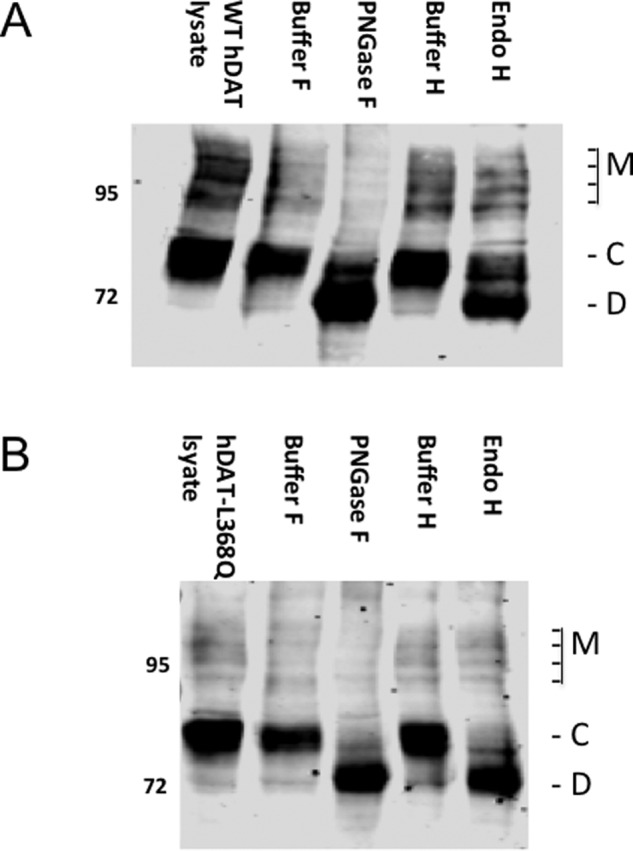
**Deglycosylation of wild-type DAT (*A*) and hDAT-L368Q (*B*) in the presence of endoglycosidase H and PNGase F.** Detergent lysates were prepared from HEK293 cells transiently expressing YFP–tagged WT hDAT (*A*) and hDAT-L368Q (*B*); aliquots thereof (15 μg) were diluted in the appropriate buffer and incubated in the absence (*second* and *fourth lane*) and presence of PNGase F (1000 units, *lane 3*) and of endoglycosidase H (1500 units, *Endo H*, *lane 5*) for 2 h at 37 °C. Thereafter, the proteins were subjected to denaturing gel electrophoresis and transferred to nitrocellulose. Immunoreactivity for DAT was visualized by immunoblotting with an antibody directed against the YFP tag. The endoglycosidase H-sensitive and -resistant bands are designate *C* (core-glycosylated) and *M* (mature, complex glycosylated), respectively; *D* denotes the deglycosylated protein.

**Figure 5.**
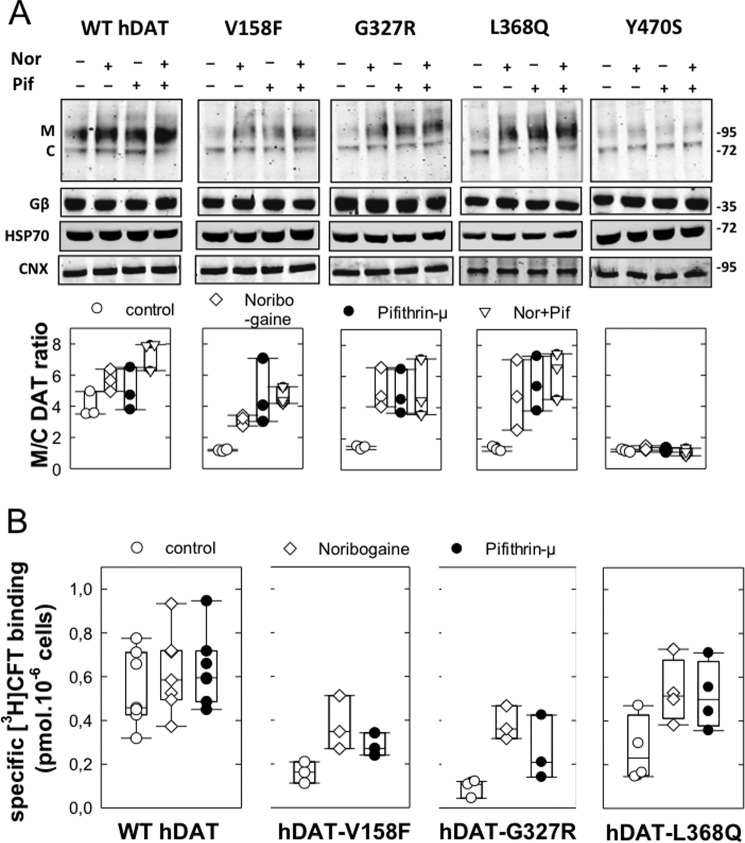
**Increase in mature, complex glycosylated species of hDAT-V158F, -G327R, and -L368Q but not of hDAT-Y470S (*A*) and of [^3^H]CFT binding to hDAT-V158F, -G327R, and -L368Q after cellular preincubation in the presence of noribogaine and pifithrin-μ (*B*).** *A*, after cellular preincubation in the absence (control lane) and presence of noribogaine (*Nor*, 30 μm), pifithrin-μ (*Pif*, 5 μm), and the combination thereof for 24 h, detergent lysates were prepared from HEK293 cells transiently expressing YFP–tagged wild-type DAT (WT, *left-hand panel*), hDAT-V158F, -G327R, -L368Q, and -Y470S as indicated. After electrophoretic separation of proteins and their transfer to nitrocellulose membranes, immunoreactivity of hDAT and of the other mutants was detected via their N-terminal tag with an antibody directed against GFP. *M* and *C* indicate the position of the mature and core glycosylated (ER resident) forms of the proteins, respectively. Lysates were also blotted for the G protein β-subunits (*G*β), HSP70-1A (*HSP70*), and calnexin (*CNX*) as a loading control for plasma membranes, cytosole, and ER membranes, respectively. The *diagrams* at the *bottom* show a quantitative assessment from three independent experiments: the immunoreactivity of the mature (*M*) and core glycosylated band (*C*) was quantified by densitometry, and the ratio *M*/*C* was plotted. The *box plots* show the median and the interquartile range; *whiskers* indicate the 5–95% confidence interval. In all instances but hDAT-Y460S, the ratio was significantly higher in cells pretreated with noribogaine, pifithrin-μ, or the combination thereof than in the corresponding untreated control cells (*p* < 0.05, Friedmann test followed by a post hoc comparison according to the Holm–Sidak method). *B*, binding of [^3^H]CFT to intact HEK293 cells transiently expressing wild-type hDAT and hDAT mutants, which had been treated with noribogaine (30 μm) or pifithrin-μ (5 μm) for 24 h prior to the binding assays. Shown are the individual values from three to six independent experiments carried out in triplicate for wild-type hDAT and hDAT-V158F, -G327R, and -L368Q and a *box plot* with the median and the interquartile range; *whiskers* indicate the 5–95% confidence interval. Binding after preincubation with noribogaine or prifithrin-μ differed in a statistically significant manner from the corresponding control binding (*p* < 0.05, Friedmann test followed by a post hoc comparison according to the Holm–Sidak method).

Only transporters located at the plasma membrane can take up substrate. Hence, specific [^3^H]dopamine uptake provides quantifies cell surface levels of DAT. If noribogaine and pifithrin-μ increased substrate uptake by pharmacochaperoning, these compounds ought to augment the export of DAT mutants from the ER and reduce their association with proteinaceous chaperones. The enhanced rate of ER export can be assessed by examining the ratio of core-glycosylated, ER-resident transporter to the mature form with complex glycans, which are acquired in the Golgi apparatus. DAT carries three glycosylation sites (23); this results in extensive microheterogeneity and thus multiple immunoreactive bands after denaturing electrophoresis (*first lane* in Fig. 4*A*). Accordingly, we determined for both the wild-type protein (Fig. 4*A*) and the mutant hDAT-L368Q after pharmacochaperoning (Fig. 4*B*), which species corresponded to the mature glycosylated form by enzymatic digestion; protein-*N*-glycosidase F (PNGase F) and endoglycosidase H remove all glycan moieties or the core glycan, respectively, to produce a deglycosylated band migrating at 72 kDa (*bands* labeled *D* in Fig. 4, *A* and *B*). Based on this analysis, we conclude that the ER-resident core-glycosylated species migrated at 80 kDa (*bands* labeled *C* in Fig. 4, *A* and *B*). The mature glycosylated, endoglycosidase H-resistant species migrated in the range of 95–110 kDa (*bands* labeled *M* in Fig. 4, *A* and *B*). This assignment allowed for exploring the action of noribogaine and pifithrin-μ; by contrast with lysates from cells expressing wild-type DAT (*left-hand blot* in Fig. 5*A*), lysates prepared from cells expressing DAT-V158F, DAT-G327R, DAT-L368Q, and DAT-Y470S contained predominantly core-glycosylated, *i.e.* ER-resident transporter (compare *first lane* in each of the *top blots* in Fig. 5*A*). The mature glycosylated bands (*M*) increased, if cells expressing the mutantsDAT-V158F, DAT-G327R, and DAT-L368Q had been pretreated with noribogaine, pifithrin-μ, or the combination thereof (*right-hand blot* in Fig. 5*A*). In contrast, these manipulations did not affect the glycosylation state of DAT-Y470S. This was predicted because neither pretreatment with pifithrin-μ nor with noribogaine enhanced uptake of dopamine in cells expressing DAT-Y470S (Fig. 2*B*).

Inhibitors bind to the outward-facing conformation, which is contingent on the presence of high Na^+^ concentrations (24). Hence, binding of the radiolabeled inhibitor [^3^H]CFT to intact cells only detects transporters at the cell surface (20). Binding of [^3^H]CFT was increased in cells expressing DAT-V158F, DAT-G327R, and DAT-L368Q, if they had been pretreated with noribogaine or pifithrin-μ (Fig. 5*B*). Taken together, these experiments verified not only that noribogaine or pifithrin-μ enhances export of the DAT mutants from the ER to the Golgi apparatus but also that this translated into their subsequent delivery to the cell surface.

**Figure 6.**
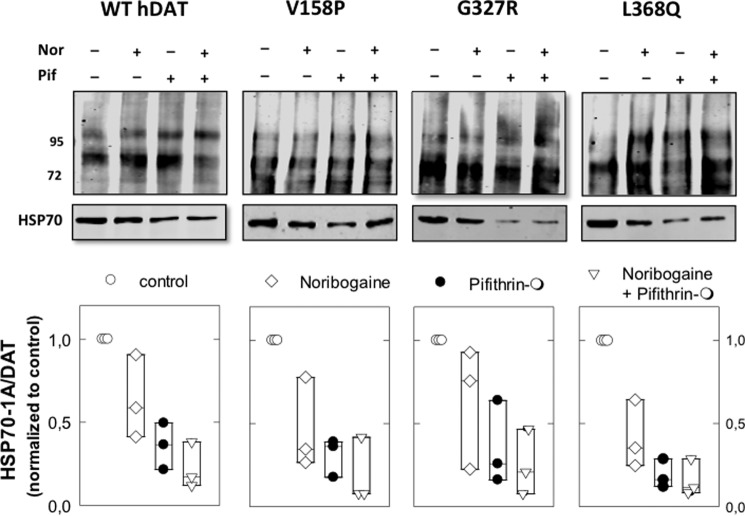
**Pharmacochaperoning of hDAT-V158F, -G327R, and -L368Q with noribogaine and pifithrin-μ reduces the amount of associated HSP70-1A.** HEK293 cells transiently expressing YFP–tagged hDAT or hDAT-V158F, -G327R, and -L368Q were incubated in the absence (*first lane* in each blot) and presence of noribogaine (*Nor*, 30 μm), pifithrin-μ (*Pif*, 5 μm), and the combination thereof for 24 h. Detergent lysates were prepared with and the transporters was immunoprecipitated with an anti-GFP antibody and immunoreactive bands were detected with appropriate antibodies directed against GFP (for DAT), and HSP70-1A as described under “Experimental procedures.” The *diagrams* at the *bottom* show a quantitative assessment from three independent experiments; the immunoreactivity of HSP70-1A was quantified by densitometry and related to the immunoreactivity of DAT. The value determined in the absence of any treatment was set 1 to normalize for interassay variations. Shown are the individual values from three independent experiments and a box plot with the median and the interquartile range. The difference between untreated cells and all other treatments was statistically significant (*p* < 0.05, Friedman test followed by a post hoc comparison according to the Holm–Sidak method).

Folding-deficient mutants of SERT are trapped in the ER in complex with the luminal chaperone calnexin and/or the cytosolic chaperone HSP70-1A (15, 16). HSP70-1A is part of the heat shock protein relay, which operates on the C terminus of SERT. It is released from the folded state to allow for recruitment of the COPII coat (8). By inference, the actions of noribogaine and pifithrin-μ are predicted to reduce the complex formation between HSP70-1A and DAT-V158F, DAT-G327R, and DAT-L368Q. We verified this prediction by immunoprecipitating these three DAT mutants from untreated control cells and from cells, which had been treated with noribogaine or pifithrin-μ. In fact, either manipulation increased the total amount of DAT-V158F, DAT-G327R, and DAT-L368Q, which was recovered by immunoprecipitation (*upper immunoblots* in Fig. 6), but it reduced the relative amount of HSP70-1A, which was found in complex with DAT-V158F, DAT-G327R, and DAT-L368Q (*lower immunoblots* and *bar diagrams* in Fig. 6). The combination of noribogaine and pifithrin-μ again resulted in the largest effect on both the total amount of DAT-V158F, DAT-G327R, and DAT-L368Q and on complex formation with HSP70-1A (*right-hand lanes* in Fig. 6).

**Figure 7.**
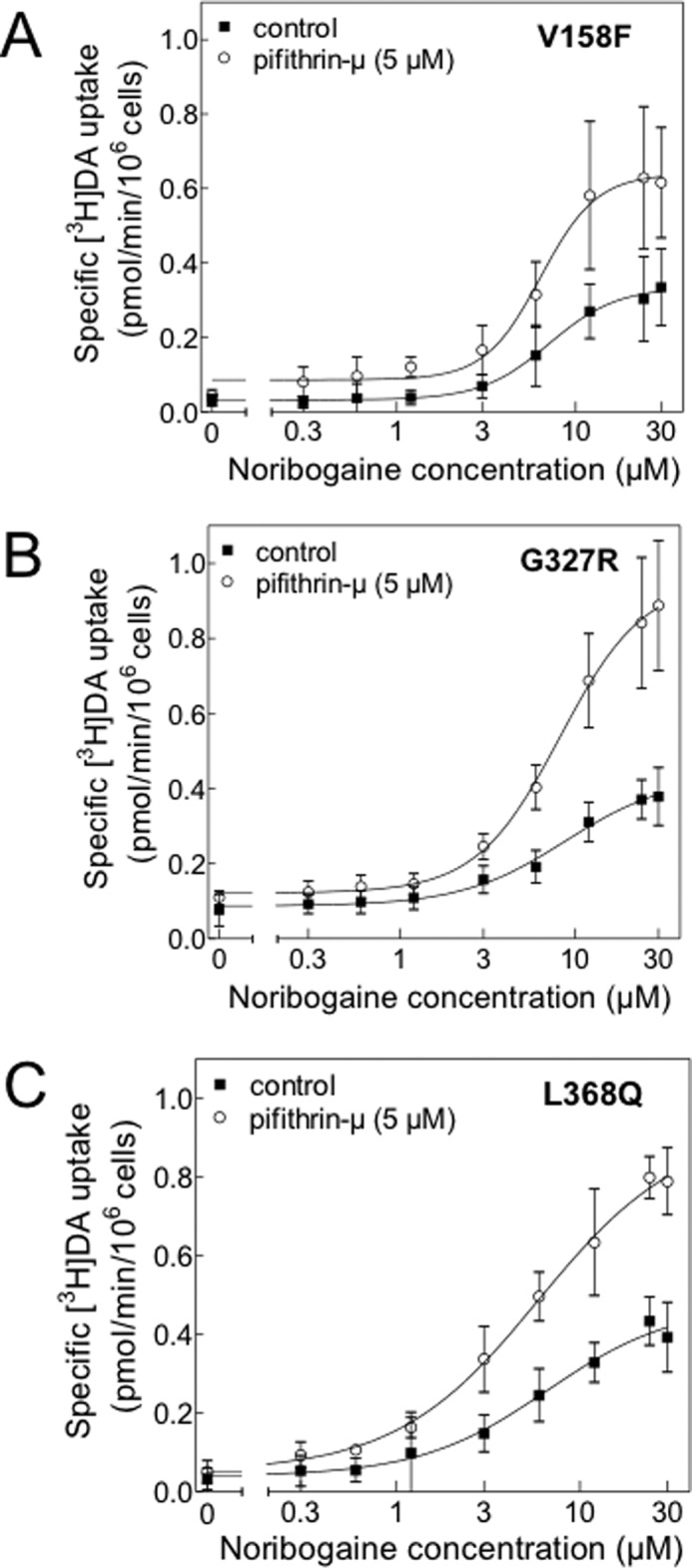
**Additive effect of pifithrin-μ on noribogaine-induced rescue of folding-deficient hDAT-V158F (*A*), hDAT-G327R (*B*), and hDAT-L368Q (*C*).** HEK293 cells transiently transfected with YFP–tagged hDAT-V158F, hDAT-G327R, and hDAT-L368Q were seeded onto 48-well plates and incubated in the absence (control, *closed squares*) and presence of pifithrin-μ (5 μm, *open circles*) with increasing concentrations of noribogaine for 24 h. Transporter function was quantified by measuring specific [^3^H]dopamine uptake. The data are from three independent experiments done in triplicate; *error bars* indicate S.D. The *curves* were generated by fitting the data points to the Hill equation. The *E*_max_ values (pmol·min^−1^·10^−6^ cells) in the absence and presence of pifithrin-μ, respectively, are for hDAT-V158F 0.33 (95% confidence interval: 0.28–0.39) and 0.64 (0.56–0.71), for hDAT-G327R 0.44 (0.34–0.53) and 0.96 (0.82–1.09), and for hDAT-L368Q 0.47 (0.36–0.59) and 0.92 (0.78–1.07), respectively. A calculation of the *Z* scores showed that the pifithrin-μ–induced increase in dopamine uptake was statistically significant in all three instances (*p* < 0.001). In contrast, pifithrin-μ did not affect the EC_50_ values for noribogaine; these EC_50_ values were for V158F 7.1 (5.1–9.9 μm) and 6.4 (5.0–8.2 μm), for hDAT-G327R 8.9 (5.4–14.7 μm) and 8.3 (6.2–10.9 μm), and for hDAT-L368Q 6.6 (3.8.0–11.6 μm) and 6.0 (3.8–11.6 μm) in the absence and presence of pifithrin-μ, respectively.

We also verified that the combination of noribogaine and pifithrin-μ elicited an additive effect by examining the rescue of substrate uptake in HEK293 cells transiently expressing hDAT-V158F (Fig. 7*A*), hDAT-G327R (Fig. 7*B*), and hDAT-L368Q (Fig. 7*C*); if these cells had been preincubated with increasing concentrations of noribogaine, [^3^H]dopamine uptake subsequently increased in a concentration-dependent manner (*closed squares* in Fig. 7). The presence of 5 μm pifithrin-μ during the preincubation enhanced the pharmacochaperoning effect of noribogaine by increasing the maximum transport velocity by ∼2-fold (*open circles* in Fig. 7). However, the EC_50_ values of noribogaine were not affected by the presence noribogaine. Thus, the two compounds acted in an additive manner, but they did not potentiate each other.

### Functional restoration of human DAT mutants in transgenic Drosophila lines

**Figure 8.**
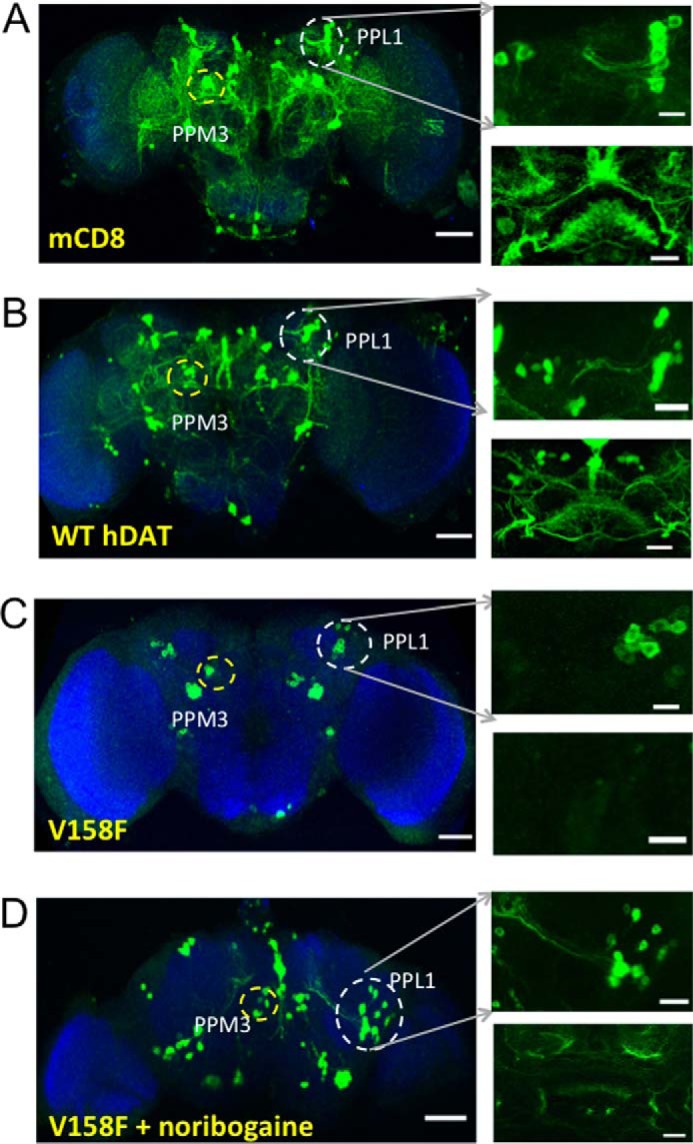
**Effect of pharmacochaperoning on the trafficking of hDAT-V158F in dopaminergic neurons of adult fly brain.** Confocal images of the posterior half of mounted adult fly brains, respectively. Neurons were visualized by staining with an antibody against neuronal cadherin (*blue background*). TH-GAL4–driven expression in neurons of mCD8-GFP (*A*), YFP-hDAT-WT (*B*), YFP-hDAT-V158F in brains from untreated (*C*), and noribogaine-treated flies (*D*) was visualized by staining with an antibody against GFP; the *left-hand panel* gives an overview, where clusters of PPL1 (dorsolateral posterior protocerebral) neurons and PPM3 (dorsomedial posterior protocerebral) neurons are marked. The *right top* and *bottom panels* show a magnified view of dorsolateral posterior protocerebral neuronal cluster (PPL1) and of the FB, respectively. Note that the wild-type hDAT enters the neurites of PPL1 cluster neurons (*B*), whereas hDAT-V158F mutant fails to enter the neuronal processes (*C*). In hDAT-V158F flies, which received 30 μm noribogaine through feeding, the mutant protein entered into neurites and was delivered to the presynaptic terminals in the FB (*D*). Each image is representative of at least 10 additional images per condition. *Scale bars* represent 50 μm for the *left panels* and 20 μm for the *right panels*.

The dopaminergic system is conserved throughout evolution; as in mammals, dopaminergic neurons play an important role in controlling locomotion and the sleep–wake cycle in flies (25). *D. melanogaster*, which are deficient in DAT because of a truncation after codon 343, are hyperactive; a conspicuous consequence is a substantial reduction of their sleep time. Hence the phenotype of the DAT-null genotype is referred to as *fumin* (Japanese for “sleepless”; Ref. 26). We created several *Drosophila* transgenic lines for wild-type hDAT and the human DAT mutants, which give rise to infantile dystonia and parkinsonism. DAT transgene expression was targeted to dopaminergic neurons using TH-GAL4. We first compared intracellular localization of wild-type and mutant transporters. Sleep regulation requires dopaminergic innervation in the fan-shaped body. This can be visualized by TH-GAL4–driven expression of GFP-tagged murine CD8, delineating both the neuronal soma arranged in distinct cell clusters and their axonal projections (Fig. 8*A*). Here, we focused specifically on the cluster of dorsolateral posterior protocerebral neurons (PPL1, *right-hand top image* in Fig. 8*A*) and their projections into the dorsal fan-shaped body (FB; *right-hand top image* in Fig. 8*A*), because they mediate the dopamine-induced silencing of sleep-promoting neurons (27). As expected, TH-GAL4–driven expression of YFP–tagged hDAT resulted in delivery of the protein to the axonal territory; thus, hDAT was enriched in the synaptic region of the FB (Fig. 8*B*). In contrast, the folding-deficient mutant hDAT-V158F was confined to the cell bodies of dopaminergic neurons and did not enter the axonal compartment (Fig. 8*C*). If flies harboring the hDAT-V158F mutant received 30 μm noribogaine via their food from the first instar larval stage up to day 15, hDAT-V158F entered into neurites and reached presynaptic sites (Fig. 8*D*).

**Figure 9.**
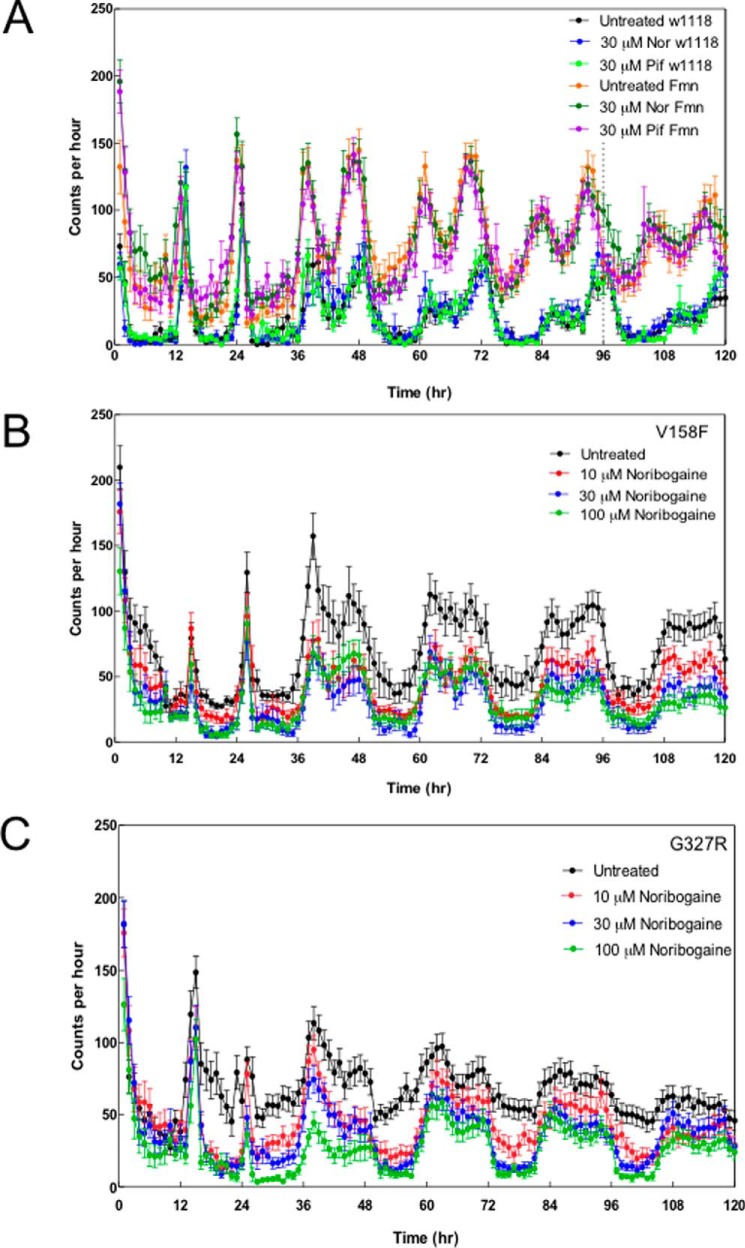
**Locomotor activity of w1118, dDAT-deficient *fumin* flies (*A*), hDAT-V158F (*B*), and hDAT-G327R flies (*C*).** Locomotor activity of individual flies was measured using a *Drosophila* activity monitoring system (TriKinetics). Beam crossings were recorded over 1-min time intervals for 7 days and combined into 60-min bins. The graph shows data from days 2–6 (on day 1 flies were allowed to recover from CO_2_ anesthesia; data from day 7 were omitted because the circadian rhythm became progressively desynchronized). Flies received the indicated concentrations of drugs in the food pellet, *i.e.* 30 μm noribogaine and 30 μm pifithrin-μ denoted by *blue* and *light green symbols* and *lines* for w1118 and *dark green* and *purple* for *fumin* (*fmn*) flies, respectively (*A*), and *red*, *blue*, and *green symbols*/*lines* for 10, 30, and 100 μm noribogaine (*B* and *C*), respectively. The data are means ± S.D. from three independent experiments, which were carried out in parallel with 10 flies/condition.

**Figure 10.**
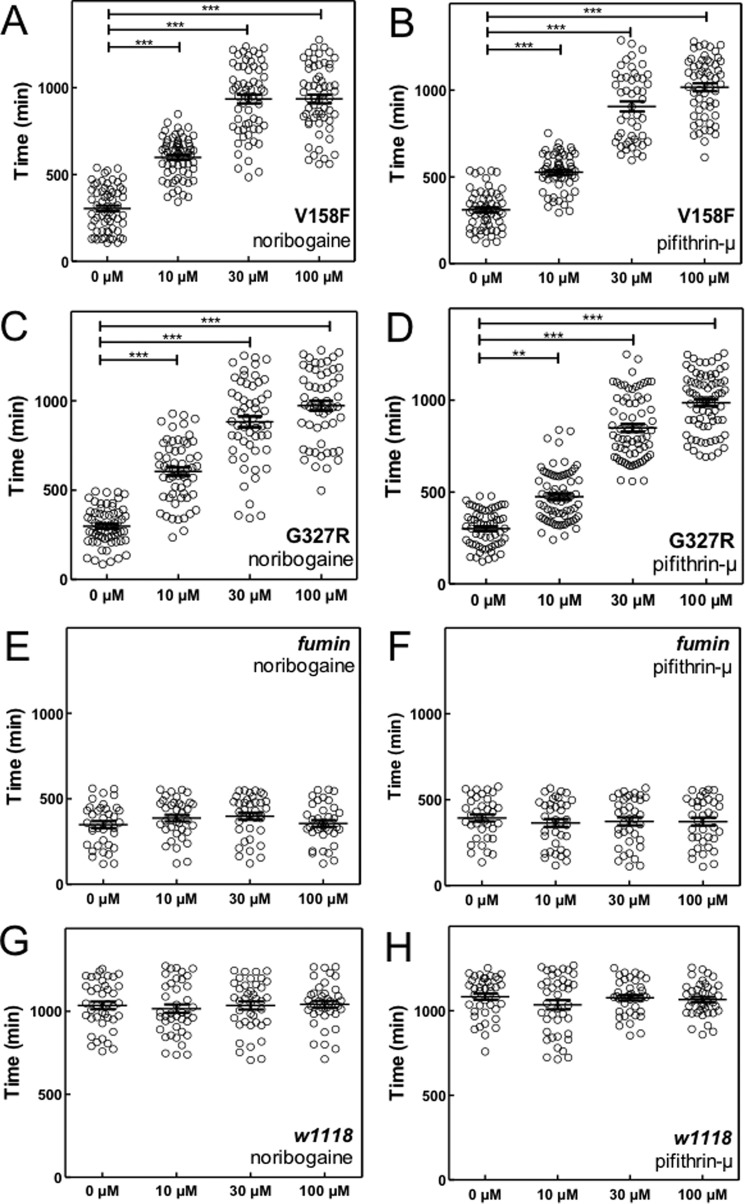
**Restoring normal sleep duration by treating flies harboring transgenic hDAT-V158F and hDAT-G327R with noribogaine and pifithrin-μ.** 3–5-day-old male flies expressing UAS-hDAT-V158F (*A* and *B*) and UAS-hDAT-G327R (*C* and *D*) in the *fumin* background received food lacking or containing the indicated concentrations of noribogaine (*A* and *C*) or of pifithrin-μ (*B* and *D*); *fumin* (*E* and *F*) and *w1118* (*G* and *H*) flies were used as controls. Total amount of sleep was calculated from activity recordings similar to those shown in Fig. 9 using pySolo as outlined under “Experimental procedures” for every condition. *Empty circles* shown in *A–H* represent recordings of individual flies, which were collected in five independent experiments using 8–15 flies/condition. The means ± S.E. are indicated. Statistical significance of the observed differences was determined by analysis of variance followed by Dunn's post hoc test (**, *p* < 0.01; ***, *p* < 0.001, significantly different from control).

The observation that pharmacochaperoning by noribogaine restored trafficking of hDAT-V158F in a DAT-deficient background to the presynaptic endings in the fan-shaped body predicted a correction of the sleeping deficit. Accordingly, adult male flies were placed individually into a transparent tube, which allowed for monitoring their activity by recording their crossing of an infrared light beam. The circadian rhythm was entrained by 2 days on a 12-h light/12-h dark cycle. Thereafter, the flies were switched to a 12-h dark/12-h dark cycle, and their activity was recorded for the next 5 days (Figs. 9 and 10). It is evident from Figs. 9*B* and 10*A* that flies harboring hDAT-V158F were hyperlocomotive and slept on average for ∼300 min/day. This was equivalent to the sleep time seen in homozygous DAT-deficient *fumin* flies (Fig. 10, *E* and *F*) and substantially shorter than the average of ∼1000 min/day of the isogenic *w1118* control strain. However, if flies expressing hDAT-V158F were fed with food fortified with increasing concentrations of noribogaine, there was a dose-dependent decrease in locomotion (Fig. 9*B*) and, hence, an increase in sleep time (Fig. 10*A*). The prolongation of sleep was also seen upon administration of pifithrin-μ (Fig. 10*B*). In fact, at the highest dose of either compound, sleep duration was restored to the level seen in *w1118* control flies. In contrast, neither hyperlocomotion (Fig. 9*A*) and the short sleep in the DAT-deficient *fumin* flies (Fig. 10*E*) nor the normal locomotion (Fig. 9*A*) sleep of *w1118* flies (Fig. 10*G*) was affected by noribogaine. Equivalent observations were made with pifithrin-μ (Fig. 10, *F* and *H*). We also examined the action of these two compounds in transgenic flies harboring other disease-causing hDAT mutants. The only responsive mutant was hDAT-G327R (Figs. 9*C* and 10, *C* and *D*). Most notably, it was not possible to test hDAT-L368Q; expression of this mutant in the developing nervous system triggered early embryonic lethality in the *fumin* background, indicating a neomorphic activity.

**Figure 11.**
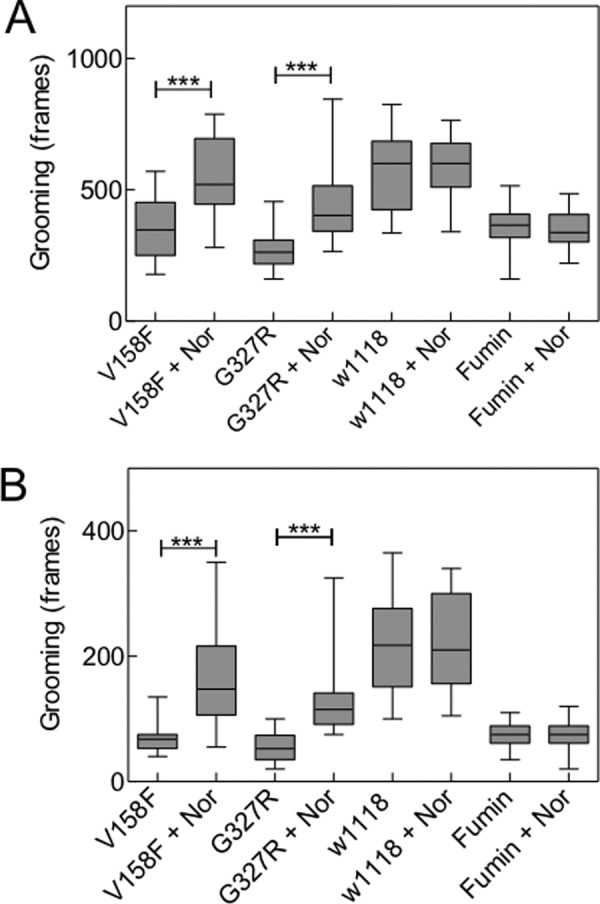
**Noribogaine treatment alters grooming behavior of hDAT-V158F and -G327R flies.** Grooming was recorded and quantified as described under “Experimental procedures.” Briefly, flies harboring hDAT-V158F and hDAT-G327R and control (w1118 and *fumin*) flies were transferred individually into a chamber, where their behavior was video recorded for a period of 5 min. Videos were converted into seven images/s; 2100 images/fly were studied for grooming of front legs, head (including eyes), abdomen, wings, and back legs. Total grooming (*A*) and head grooming (*B*) were calculated for untreated and noribogaine (*Nor*)-treated flies. *Box plots* show median and interquartile range, and *whiskers* indicate the minimum and maximum range. Statistical analysis was performed by a Kruskal–Wallis test followed by Dunn's post hoc test (*n* = 20 per genotype; ***, *p* < 0.001, significantly different from control).

Dopamine receptor activation elicits grooming in flies (28–30). Grooming is reduced in *fumin* flies; in addition, when introduced into a novel environment, *fumin* flies display substantially reduced head grooming in the first 5 min (31). Accordingly, we also evaluated grooming of flies by recording their behavior in a novel environment over the initial 5-min period. Similar to *fumin* flies, flies harboring hDAT-V158F and hDAT-G327R spent less time with grooming than w1118 flies (Fig. 11*A*). Consistent with previous observations (31), the difference was mainly accounted for by reduced head grooming (Fig. 11*B*). There was no appreciable difference, if grooming of other body parts was examined (not shown). Most importantly, administration of noribogaine to flies harboring hDAT-V158F and hDAT-G327R prolonged the time spent with grooming (Fig. 11, *A* and *B*). In contrast, noribogaine affected grooming neither by w1118 flies nor by *fumin* flies (Fig. 11, *A* and *B*). Dopaminergic control of grooming relies on neurons other than those that control sleep (28–30). Hence, these observations suggest that the pharmacochaperoning action of noribogaine suffices to correct global dopaminergic transmission in the central nervous system of flies harboring the folding deficient mutants hDAT-V158F and hDAT-G327R.

## Discussion

The basic elements of dopaminergic neurotransmission have been conserved through evolution from flies to people (32). This is true in particular for the vesicular monoamine transporter and the dopamine transporter DAT, which operate in a relay to terminate dopaminergic signaling and replenish vesicular stores. Accordingly, flies are also susceptible to the dopamine depleting actions of the vesicular monoamine transporter inhibitor reserpine (28, 33, 34) and the stimulating actions of the DAT inhibitor cocaine (26, 36) and the dopamine releaser methamphetamine (37). It is therefore reasonable to assume that the machinery, which is required for folding and targeting of DAT, is also conserved in *D. melanogaster*. We previously provided a proof of principle by showing that it was possible to pharmacochaperone dDAT-G108Q, a folding-deficient mutant of *D. melanogaster* DAT (19, 20). Here we systematically investigated a collection of 13 independent DAT mutants associated with infantile dystonia and parkinsonism (5–7) for their responsiveness to noribogaine and pifithrin-μ. We identified two mutations that were unequivocally amenable to pharmacological rescue, *i.e.* hDAT-V158F and hDAT-G327R. This conclusion is based on the following observations: (i) consistent with their defective folding, these two DAT mutants accumulated as core-glycosylated proteins within transiently transfected HEK293 cells in stalled complexes with HSP70; (ii) preincubation with noribogaine and pifithrin-μ resulted in the release of HSP70 and in enhanced ER export such that the proteins acquired the Golgi-derived complex glycosylation and reached the cell surface; (iii) accordingly, this pretreatment also restored substrate uptake by hDAT-V158F and hDAT-G327R to appreciable levels; and (iv) most importantly, noribogaine was also effective *in vivo*; in flies, which harbored these mutants, ingestion of noribogaine restored sleep time to normal levels. Several arguments support the conclusion that the salutary effect of noribogaine was related to its pharmacochaperone action rather than an off-target effect; noribogaine did not enhance sleep in *fumin* flies, in which the endogenous dDAT is truncated after TM6 and is thus non-functional (26). Noribogaine also did not affect sleep in *w1118* flies, *i.e.* in the presence of a functional endogenous DAT. Finally, noribogaine restored trafficking of hDAT-V158F from the neuronal soma to the axonal territories, thus providing independent evidence for its pharmacochaperoning activity. The therapeutic implications of our findings are self-evident; phase I trials show that noribogaine is reasonably well tolerated up to doses of 180 mg/day by adults (38, 39). A trial of noribogaine may be justified in children suffering infantile dystonia and parkinsonism, which is due to the mutations hDAT-V158F and hDAT-G327R. In flies harboring these mutants, the HSP70 inhibitor pifithrin-μ was also effective in restoring sleep. However, the therapeutic implications of these observations are less obvious than for noribogaine. Pifithrin-μ has not yet been used in people. It is worth noting that in all hDAT mutants that were responsive to noribogaine, we observed an additive action of pifthrin-μ. A previous analysis of folding-deficient SERT mutants showed that all possible effects were elicited by the combination of noribogaine and pifithrin-μ; depending on where each individual mutant was stalled in the folding trajectory, pifithrin-μ potentiated the pharmacochaperoning action of noribogaine by shifting the concentration-response curve of noribogaine to the left and by increasing the maximum transport rate did not have any appreciable effect or shifted the concentration-response curve of noribogaine to the right but increased the maximum transport rate (15). Here, we uniformly observed an additive effect in all three DAT mutants, which indicated that noribogaine and pifithrin-μ acted in an independent manner. The folding of polytopic membrane proteins is poorly understood (8). However, pharmacochaperoning provides a glimpse of the folding trajectory (19). Our observations indicate that (i) the three DAT mutants are stalled at similar positions of the folding trajectory and (ii) that progress was limited by two separate energy barriers that were susceptible to the action of noribogaine and of pifthrin-μ, respectively.

It is worth noting that hDAT-L368Q was rescued by pharmacochaperoning in cell culture, but that we could not verify the action of noribogaine in flies, because flies expressing hDAT-L368Q did not hatch. The underlying cause is unknown, but the accumulation of the misfolded protein may lead to ER stress and thus eliminate dopaminergic neurons during development. Loss of dopaminergic neurons in the larval stage can be lethal (40). The concomitant presence of the truncated dDAT provides a possible explanation regarding why the accumulation of misfolded hDAT-L368Q is more detrimental for dopaminergic neurons than for HEK293 cells: SLC6 transporters from oligomers (41–43). Oligomerization occurs in the ER (44), and fragments including half-molecules suffice to support oligomer formation in the ER (12). It is therefore conceivable that the folding problem of hDAT-L368Q is aggravated in *Drosophila* dopaminergic neurons, because it associates with the endogenous *fumin* fragment of dDAT, and it is this complex that causes the toxicity. We do not see this expression of human mutants in the *fumin* background as a limitation; in fact, in infantile dystonia and parkinsonism, the vast majority of individuals are compound heterozygotes (5–7). Thus, pharmacochaperoning must rescue a responsive mutant in the presence of a second folding-deficient mutant, which is encoded by the second allele. In our *Drosophila* experiments, the presence of the *fumin* fragment of dDAT is likely to have mimicked this situation and increased the stringency of the pharmocochaperoning experiment. In a recent study, four disease-causing hDAT mutants (*i.e.* hDAT-L224P, -A314V, -R445C, and -P529L) were examined for their responsiveness to pharmacochaperoning; pretreatment of HEK293 cells with by ibogaine and bupropione restored surface expression of and substrate uptake by hDAT-A314V and of hDAT-R445C (45). We have not been able to recapitulate these findings; it is evident from Fig. 2 that preincubation of cells with noribogaine did not restore uptake by hDAT-A314V to any appreciable level and only very modestly stimulated uptake by hDAT-R445C. We do not consider it likely that this discrepancy can be attributed to our using noribogaine, because noribogaine is more potent than ibogaine on DAT (46). It appears more likely that the discrepancy can be accounted for by the different transfection regimen; we assessed pharmacochaperoning in transiently transfected cells, and Beerepoot *et al.* (45) used stably transfected cells. Clonal selection may magnify a pharmacochaperoning effect, which escapes detection when assessed in the entire population. At the very least, this discrepancy highlights the limitations of relying solely on cell culture experiments to assess pharmacochaperoning. It is also not without precedent; discrepancies in HEK293 cell-based assays account for the equivocal outcome of the phase III trial of migalastat, a substrate analogue, which is capable to pharmacochaperone some folding-deficient mutants of lysosomal α-galactosidase in Fabry's disease (47).

Folding diseases require a personalized approach; mutants are stalled at different points in the folding trajectory (14, 15). It is therefore not surprising that only a fraction of the tested hDAT mutants were rescued by noribogaine. Thus, it is safe to conclude that many different compounds will be required, but there are several reasons to justify an optimistic outlook. First, the monoamine transporters DAT has a very rich pharmacology; the chemical space has been and is being explored for potential ligands, in part by illicit commercial activities (48). In fact, we recently identified a compound with pharmacochaperoning activity by analyzing a series of partial substrates (49). Second, the current study shows that *D. melanogaster* can be used as a model system to test candidate pharmacochaperones within a reasonable time frame; *Caenorhabditis elegans* is also being explored as an alternative model organism (50, 51). Third, an additional class of compounds can be envisaged by analogy with the cystic fibrosis transmembrane conductance regulator (CFTR/ABCC7), where the corrector lumacaftor remedies the folding deficiency of some mutants (52) and the potentiator ivacaftor potentiates the action of cAMP on channel gating and thus enhances their Cl^−^ conductance (53). We previously noted that after pharmacochaperoning-induced correction of the folding deficit, dDAT-G108Q displayed a reduced affinity for substrate (20). This was also seen with the three mutants, which were responsive to pharmacochaperoning regardless of whether the correction was achieved by noribogaine or by pifitrin-μ. This is not unforeseen and can be rationalized as follows. Each of the mutations impeded the conformational transitions required for progression through the folding trajectory. Accordingly, these mutations are also likely to affect the conformational transitions that underlie the transport cycle. By analogy with CFTR, we therefore predict that in some instances, a combination of pharmacochaperone/corrector and allosteric activator/potentiator may be required to remedy the transport deficiency. DAT has an allosteric metal-binding site, occupancy of which can result in both stimulation and inhibition of the transport cycle (54, 55). Thus, allosteric activation of DAT mutants is, in principle, conceivable.

## Experimental procedures

### Chemicals

Noribogaine was obtained from Cfm Oskar Tropitzsch GmbH (Marktredwitz, Germany). Pifithrin-μ was obtained from Sigma–Aldrich. [^3^H]Dihydroxyphenylethylamine (dopamine; 36.6 Ci/mmol) and [^3^H]CFT (76 Ci/mmol) were obtained from PerkinElmer Life Sciences. Cell culture media, supplements, and antibiotics were purchased from Invitrogen. Other cell culture reagents, such as BSA and Complete^TM^ protease inhibitor mixture, were purchased from Roche Applied Science, SDS from BioMol (Hamburg, Germany), scintillation mixture (Rotiszint® eco plus), and Tris from Carl Roth (Karlsruhe, Germany). Anti-GFP antibody (ab290) was obtained from Abcam (Cambridge, UK). Protein A-Sepharose and horseradish peroxidase–linked anti-rabbit IgG1 antibody were purchased from Amersham Biosciences. All other chemicals used in experiments were of analytical grade. The 0.4% trypan blue solution was obtained from Sigma–Aldrich.

### Drosophila genetics

The transgenic UAS reporter lines for YFP–tagged hDAT-R85L, V158F, L224P, G327R, L368Q, P554L, and wild-type hDAT were generated using the pUAST-attB vector and injected into embryos of ZH-86Fb flies (Bloomington stock no. 24749). The landing site on the third chromosome (3R 86F) was selected for two reasons. First, because the gene encoding the dopamine transporter is located on the second chromosome, hDAT mutants could be expressed in a dDAT-deficient background. Second, the landing site showed a high transgene integration rate and leaky expression in our previous study (20). All flies were kept at 25 °C in a 12-h light/12-h dark cycle, and all crosses were performed at 25 °C. TH-Gal4 and UAS mCD8:GFP were ordered from the Bloomington Drosophila stock center (Bloomington, IN); *w1118* and *fumin* flies used in our experiments were described previously (26). The genotypes of flies used for confocal imaging were *w1118*; +/+; *TH-Gal4/UAS-mCD8GFP* (Fig. 7*A*), *w1118*; +/+; *TH-Gal4/UAS-hDAT* (Fig. 7*B*), and w1118; +/+; *TH-Gal4/UAS-hDAT-V158F* (Fig. 7, *C* and *D*). For behavior, homozygous lines were used in *fumin* background. The genotypes were w1118/y; *fmn; UAS-hDAT-V158F* (Fig. 8, *A* and *B*), w1118/y; *fmn*; *UAS-hDAT-G327R* (Fig. 8, *C* and *D*), *w1118/y; fnm*(w; roo{}DAT^fmn^); +/+ (Fig. 8, *E* and *F*), and *w1118* (Fig. 8, *G* and *H*). w1118, hDAT mutant and *fumin* flies were isogenized by crossing with balancer flies (Bloomington stock no. 3704).

### Mutagenesis, cell culture, and transfection

A QuikChange Lightning site-directed mutagenesis kit (Agilent Technologies, Santa Clara, CA) was used to introduce mutations into plasmids encoding YFP-hDAT. A QuikChange primer design tool provided by the manufacturer was used to design primers. HEK293 cells were grown at 37 °C, in a 5% CO_2_ humidified atmosphere, in DMEM, supplemented with 10% fetal calf serum, penicillin (60 mg/liter), and streptomycin (100 mg/liter). 24 h after seeding, the cells were transiently transfected with plasmids (0.5–2 μg/10^6^ cells) using Lipofectamine 2000 (Life Technologies, Carlsbad, CA) according to the instructions of the manufacturer.

### Radiotracer assays and confocal microscopy

Uptake assays were performed using transfected HEK293 cells. Briefly, cells were seeded onto poly-d-lysine coated 48-well plates, to a density ∼10^5^ cells/well. 24 h after seeding, the cells were washed with Krebs–Ringer–HEPES buffer (10 mm HEPES, 120 mm NaCl, 3 mm KCl, 2 mm CaCl_2_, 2 mm MgCl_2_, 2 mm glucose monohydrate, 1 mm tropolone, 10 μm pargyline, and 1 mm ascorbic acid, pH 7.3) and incubated with 0.2 μm [^3^H]dopamine at room temperature for 5 min. Ice-cold Krebs–Ringer–HEPES buffer was used to terminate the uptake. For experiments involving *K_m_* and *V*_max_ measurements, [^3^H]dopamine was diluted with unlabeled dopamine to obtain final concentrations of 0.2, 1, 3, 10, 15, and 30 μm. Background uptake was determined in the presence of 30 μm mazindol (20).

For confocal imaging, HEK293 cells expressing wild-type YFP-hDAT or mutants were seeded onto poly-d-lysine–coated ibidi® glass-bottomed chambers. Cell imaging was performed 24 h after seeding using a Zeiss LSM780 equipped with an argon laser (at 30 milliwatts) and a 63× oil immersion objective (Zeiss Plan-Neofluar). Trypan blue solution was used to label the plasma membrane.

### Enzymatic deglycosylation

HEK293 cells were lysed 24 h after transfection in a buffer containing 50 mm Tris·HCl (pH 8.0), 150 mm NaCl, 1% dodecylmaltoside, 1 mm EDTA, and Complete^TM^ protease inhibitor mixture; insoluble material was removed by centrifugation (30 min at 13,000 × *g* at 4 °C). Aliquots of the lysate (15 μg of protein) were denatured by heating to 100 °C for 10 min in a solution containing 0.5% SDS and 40 mm DTT; subsequently, the sample was diluted in 50 mm Na_2_HPO_4_/NaH_2_PO_4_ (pH 7.5), 1% Nonidet P-40, or 50 mm CH_3_COO·Na (pH 6.0) and digested with peptide-*N*-glycosidase F (1000 units) or endoglycosidase H (1500 units), respectively, for 2 h at 37 °C according to the manufacturer's instructions. Proteins were separated on SDS-PAGE (8%), blotted, and visualized with anti-GFP antibody as described above.

### Co-immunoprecipitation

Immunoprecipitation was performed as described previously (14, 15). Briefly, hDAT and mutant transfected cells were washed twice with PBS, mechanically detached in lysis buffer (50 mm Tris·HCl, pH 8.0, 150 mm NaCl, 1% dodecylmaltoside, 1 mm EDTA, and the Complete^TM^ protease inhibitor mixture) and collected by centrifugation. Further centrifugation (16,000 × *g* for 30 min at 4 °C) was performed to remove detergent-insoluble material. Equal amounts of protein (2 mg/sample) were incubated with 4 μl of anti-GFP antibody (20 μg of IgG) for 16 h. Thereafter, pre-equilibrated protein A-Sepharose (20 mg protein A/sample) was added and incubated for 5 h on shaker at 4 °C. The beads were collected by centrifugation and washed with lysis buffer three times. Proteins were eluted in 0.1 ml of sample buffer containing 40 mm dithiothreitol and 1% mercaptoethanol at 45 °C for 30 min. An aliquot from the original cellular lysate was also denatured in sample buffer. The aliquots were resolved by polyacrylamide gel electrophoresis, and proteins were transferred onto nitrocellulose membranes. The membranes were blocked with 5% bovine serum albumin in Tris-buffered saline supplemented with 0.1% Tween 20. Membranes were then incubated with anti-GFP, anti-calnexin, or anti-HSP70-1A antibody overnight at 4 °C. The membranes were washed in buffer and incubated with HRP-conjugated secondary antibody (1:5000). The immunoreactivity was detected by chemiluminescence.

### Immunohistochemistry and imaging

Adult fly brains were dissected in PBS and fixed in 2% paraformaldehyde in PBS for 1 h at room temperature. Subsequently, brains were washed in 0.1% Triton X-100 in PBS (PBST) three times for 20 min and blocked in 10% goat serum for 1 h at room temperature. The brains were then incubated in antibodies directed against GFP (1:1000 dilution; A-11122, Invitrogen) and neuronal caedherin (1:20, DSHB) overnight in 10% goat serum at 4 °C. Thereafter, the brains were washed three times in PBST for 20 min before incubating with Alexa Fluor 488 goat anti-rabbit IgG (1:500; Invitrogen) and Alexa Fluor 647 goat anti-mouse IgG (1:500; Invitrogen) secondary antibodies in 10% goat serum for 3 h. The brains were washed three times with PBST and were mounted using Vectashield® (Vector Laboratory, Burlingame, CA). Images were captured on a Leica SP5II confocal microscope with 20-fold or 63× magnification. *Z*-stack images were scanned at 1.5-μm section intervals with a resolution of 1024 × 1024 pixels. Images were processed using ImageJ.

### Behavioral assays and treatments of flies

Locomotion of 3–5-day-old male flies was recorded as described previously (20) using the DAM2 *Drosophila* activity monitor system (Trikinetics, Waltham, MA). 8–15 flies were used per genotype in five independent experiments: *w1118*; *fmn*; *UAS-hDAT-V158F* and *w1118*; *fmn*; *UAS-hDAT-G327R* flies were used for behavioral assay as we observed leaky expression of the protein. The leaky expression of protein was considerably lower than the GAL4-induced overexpression, and therefore we did not use TH-GAL4 for the behavioral assay. We used *fumin* and *w1118* flies as control. Individual flies were housed in 5-mm-diameter glass tubes, which contained the food pellet supplemented with the indicated concentrations of noribogaine or pifithrin-μ, which were freshly dissolved in water and mixed with regular food (5% sucrose in 1% agarose). The flies were kept on a 12-h light/12-h dark cycle for the first 2 days, and the cycle was shifted to a 12-h dark/12-h dark for 5 subsequent days. The locomotion activity was constantly monitored during this period, and data from the second day of the dark/dark phase were used for calculations. Activity was measured in 1-min bins using software provided by the manufacturer, and the pySolo software (35) was used to quantify fly sleep. Inactivity of a fly for 5 min or more was considered as sleep. The flies received normal food (5% sucrose in 1% agarose) prior to their individual housing.

Grooming was monitored according to Ref. 31 with minor modifications. Briefly, a chamber was constructed with 25 mm in diameter and 2.9 mm in height comprising a transparent polystyrene top, acrylic bottom, and opaque lateral walls. The camera was mounted onto a compound microscope to record fly activity. 3-day-old male *w1118, fmn*, *hDAT-V158F* and *-G327R* flies received either regular food or food containing 100 μm noribogaine for 7 days. Individual flies were then transferred to the chamber using an aspirator, and their behavior was immediately recorded for a period of 5 min at 60 frames/s. Adobe Photoshop CS5 was used to convert videos recorded at 1280 × 720 resolution (60 frames/s) into a 720 × 480 JPEG2000 image sequence at a frame rate 7 frames/s. This sequence of 2100 images (7 images/s over 300 s) per fly was studied to quantify grooming behavior by an observer blinded to genotype and treatment. If a fly was observed to visibly clean its head, legs, wings, or its thorax/abdomen over at least five consecutive images (*i.e.* >0.7 s), this was scored as grooming behavior. The total duration of grooming was obtained from the sum of all grooming events. The grooming behavior was further classified into head/eye, front legs, abdomen, hind legs, and wings grooming. All behavioral experiments were performed at 25 °C.

## Author contributions

M. F., T. H., and S. S. designed the experiments; M. F., H. M. M. A., and A. K. wrote the paper. H. M. M. A. performed the experiments in Figs. 1–6 with the assistance of S. S. and A. E.-K. A. E.-K. performed the experiments in Fig. 7 with the assistance of H. M. M. A. A.K. performed the experiments in Fig. 8 with the assistance of M. S., A. E.-K., T. H., and S. S. provided reagents, advice, and cell lines. All authors reviewed the results and approved the final version of the manuscript.

## Footnotes

5 The abbreviations used are:

DAT: dopamine transporter
17-DMAG: 17-dimethylaminoethylamino-17-demethoxygeldanamycin
[^3^H]CFT: [^3^H]2-β-carbomethoxy-3-β-(4-fluorophenyl)-*N*-methyltropane
dDAT: *Drosophila* DATR
hDAT: human DAT
ER: endoplasmic reticulum
HSP: heat shock protein
SERT: serotonin transporter
PNGase F: peptide-*N*-glycosidase F
SLC: solute carrier
TH-GAL4: tyrosine hydroxylase promoter–driven yeast transcription factor GAL4
UAS: upstream activating sequence
FB: fan-shaped body.
